# Social Behaviour Drives Gut Microbiome Community Assembly Among Wild Long‐Tailed Macaques ( *Macaca fascicularis* ) in Thailand

**DOI:** 10.1002/ece3.74612

**Published:** 2026-11-01

**Authors:** Krishna Balasubramaniam, Jishnu Kesh, Nalina Aiempichitkijkarn, Robert Atwill, Suchinda Malaivijitnond, Brenda McCowan, Juthamas Nimnual, Vorthon Sawaswong, Suthida Visedthorn, Sunchai Payungporn

**Affiliations:** ^1^ Faculty of Science and Engineering, School of Life Sciences Anglia Ruskin University Cambridge UK; ^2^ Department of Biological Sciences Indian Institute of Science Education and Research (IISER) Mohali India; ^3^ School of Biology, Institute of Science Suranaree University of Technology Nakhon Ratchasima Thailand; ^4^ Department of Population Health and Reproduction, School of Veterinary Medicine University of California—Davis Davis California USA; ^5^ Department of Biology, Faculty of Science Chulalongkorn University Bangkok Thailand; ^6^ National Primate Research Center of Thailand Chulalongkorn University Saraburi Thailand; ^7^ Center of Excellence in Systems Microbiology, Department of Biochemistry, Faculty of Medicine Chulalongkorn University Bangkok Thailand; ^8^ Department of Biochemistry, Faculty of Science Mahidol University Bangkok Thailand

**Keywords:** individual behaviour, joint species distributions models, microbial communities, nonhuman primates, social interactions, social microbiome

## Abstract

The ‘social microbiome’ conceptualises gut microbial communities as ‘islands’ of host social organisation. Yet, in comparison to broader aspects of social organisation such as animals' group membership and composition, the impact of inter‐individual differences in sociality on microbiome community ecology remains less well studied. Among a wild but human‐provisioned group of long‐tailed macaques ( *Macaca fascicularis* ), we assessed the relative effects of individuals' sociality, compared to their demographic characteristics, anthropogenic and abiotic environmental factors, and microbe‐microbe associations, on the relative abundance and co‐occurrence of bacterial taxa. Bipartite network analysis showed that bacterial communities among macaques were nested rather than segregated or modular structures. Several ‘core’ bacterial genera were found across all macaques, but other ‘non‐core’ bacteria were found only in a few macaques suggesting that their occurrence/abundance may be influenced by transient environmental factors. Joint Species Distributions Models showed that macaques' sociality, particularly their dominance rank and their connectedness or centrality within their social grooming network, had the strongest impact on taxa abundance, followed by their demographic characteristics, and finally their exposure to both anthropogenic and abiotic environmental factors. Phylogenetically distant taxa were more likely to co‐occur within the same macaque hosts than phylogenetically related taxa. These findings confirm that microbiome communities are strongly influenced by the social status and connectedness of individual hosts. Nevertheless, the finding that some taxa were more prevalent among high‐ranking or well‐connected macaques, while others were more prevalent among low‐ranking or socially peripheral macaques, warrants further investigation into how individual differences in social microbiomes might affect their health and fitness. Future studies, through the collection of behavioural and microbial data on additional macaque groups, may provide further insights into the relative effects of animals' social organisation and social behaviours on microbiomes.

## Graphical abstract

Among wild long‐tailed macaques in Thailand, we tested the relative effects of individuals' sociality, compared to their exposure to environmental factors, and their demographic characteristics, on the relative abundance and assembly of gut microbiota. We found strong effects of both dominance rank and social network connectedness on gut bacterial abundance. Many phylogenetically distant bacteria also co‐occurred among the same macaque hosts. This suggests that sociality may combine with bacterial phylogenetics to influence macaque microbiomes.

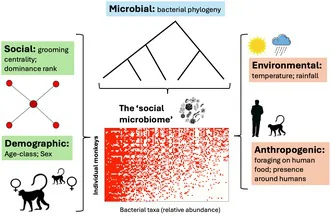

## 1 Introduction

Understanding the ecological and evolutionary underpinnings of wildlife microbiomes is an important endeavour for biologists. Aside from their widely accepted impacts on host health and survival (Kappeler et al. 2015; Sarkar et al. 2024), ecologists also contend that microbes are critical components of ecosystems that are naturally embedded within host social organisational systems (French and Holmes 2020; Ladau and Eloe‐Fadrosh 2019; Sarkar et al. 2020). Among humans and other group‐living animals, for instance, aspects of social organisation such as group‐membership and sizes, philopatry and dispersal tendencies, and kinship composition can all have profound, variant and at times complex effects on the acquisition, assembly and community structure of microbiota (Archie and Tung 2015; Baniel and Charpentier 2024; Debray et al. 2024; Sarkar et al. 2020). Of late, this has led to conceptualisation of the ‘social microbiome’. Grounded in metacommunity theory, the social microbiome concept treats bacterial taxa that constitutes the microbiome as species assembled within individual animals that may be thought of as ‘islands’ (as in island biogeography: MacArthur and Wilson 1967) that live in social groups and populations that can be thought of one or more ‘archipelagos’ of islands (Baniel and Charpentier 2024; Debray et al. 2024; Sarkar et al. 2020).

In comparison to aspects of social organisation, there is still a limited understanding of how inter‐individual differences in social behaviour impact microbiomes (Baniel and Charpentier 2024; Debray et al. 2024; Sarkar et al. 2020). While social organisation include the composition and sizes of groups, social behaviour emerges from the patterning and distribution of competitive (e.g., aggression, submissive signalling: de Vries et al. 2006; Fedigan 1983; Gammell et al. 2003) and cooperative (e.g., social or allogrooming, support during conflicts: Henzi and Barrett 1999; Kappeler and Van Schaik 2002; McFarland 2018) social interactions between individuals within groups (Hinde 1976; Kappeler and Van Schaik 2002). There is now a growing body of research that has established links between aspects of social organisation and microbiomes, for instance the effects of group composition and membership, group sizes and range‐use overlap and hosts' dispersal and immigration tendencies, on microbiome composition and turnover (reviewed in Baniel and Charpentier 2024; Debray et al. 2024; Sarkar et al. 2020). However, assessments of inter‐individual differences in social behaviour on microbiomes have shown inconsistent findings. For instance, animals' dominance ranks have been associated with greater overall microbial diversity in Cichlid fish ( *Astatotilapia burtoni* : Singh et al. 2019), but not so among spotted hyenas ( *Crocuta crocuta* : Heitlinger et al. 2017). Likewise, the connectedness or centrality of individuals within their social affiliation networks has often been associated with greater bacterial acquisition or transmission (e.g., rhesus macaques, *Macaca mulatta* : Balasubramaniam et al. 2019; yellow baboons, *Papio cynocephalus* : Tung et al. 2015; Grant's gazelles, *Nanger granti* : Brown et al. 2024), but not always with greater bacterial diversity (e.g., in yellow‐bellied marmots, *Marmota flaviventer*: Pfau et al. 2024).

One reason for such inconsistent findings could be that the afore‐reviewed studies have predominantly focused on evaluating overall microbial diversity, or their connectedness within microbial sharing or transmission networks (reviewed in Baniel and Charpentier 2024; Debray et al. 2024; Sarkar et al. 2020). In reality, social interactions might impact bacterial acquisition in complex ways; for instance, they could lead to an increase in the abundance of some bacterial taxa on account of social contact‐mediated transmission, but have the opposite or no effect on others on account of animal‐specific characteristics or their exposure to other more prominent environmental factors (Sarkar et al. 2024). Related to the latter, empirical studies of the ‘social microbiome’ need to disentangle the impact of social behaviour from other factors that influence bacterial assemblages, such as animal‐specific demographic, physiological and health‐related characteristics, asocial environmental factors such as climate, seasonal variation, foraging strategies, human or anthropogenic impact, and microbe‐specific factors such as phylogenetic relatedness and within‐host ecological associations, on bacterial assemblages (Baniel and Charpentier 2024; Debray et al. 2024; Sarkar et al. 2020). Macroecological Joint Species Distributions Models (JSDMs) can be powerful, quantitative tools to address these issues (Ovaskainen et al. 2017; Pollock et al. 2014; Tikhonov et al. 2020). Traditionally used in macroecology to assess environmental effects on species diversity and sympatry, JSDMs capture co‐variation between the presence‐absence or relative abundance of taxonomic units and features of their environment, host‐specific traits and microbial phylogenetic relationships. Pertinently, JSDMs have been successfully implemented in assessing the effects of social organisation on within‐host microbe‐microbe associations and co‐occurrence; for instance, on the co‐occurrence of protozoan endoparasites and pathogenic bacteria among African lions ( *Panthera leo* : Fountain‐Jones et al. 2019) and rodents (Mariën et al. 2022), Picobirnavirus taxonomic units among rhesus macaque groups and populations (Balasubramaniam, Navarrete Macias, et al. 2025), and finally gut microbiome assemblages and co‐occurrence in Neotropical birds (Björk et al. 2018) and meerkats (Balasubramaniam, Muller‐Klein, et al. 2025). Thus, JSDMs offer tremendous scope when it comes to advancing knowledge of social microbiomes.

Addressing the above gaps, here we assessed the impact of inter‐individual differences in animal sociality and social behaviour on the composition and community structure of the social microbiome. To realise this objective we studied wild long‐tailed macaques ( *Macaca fascicularis* ) living in human‐impacted or anthropogenic landscapes in Thailand as a model system (Gumert 2011; Malaivijitnond and Hamada 2008). Long‐tailed macaques are the most widely distributed of all macaque species in South‐East Asia, and the third most widely distributed primate species after humans and rhesus macaques (Gumert 2011; Malaivijitnond and Hamada 2008). They live across a wide range of habitats, including anthropogenic landscapes like urban and peri‐urban, agricultural and fragmented forest landscapes (Gumert 2011; Malaivijitnond and Hamada 2008). Throughout their range, long‐tailed macaques show broadly similar social organisation: they live in multi‐male multi‐female social groups in which females tend to be philopatric and males tend to disperse (Gumert 2011; Malaivijitnond and Hamada 2008; Sterck and Steenbeek 1997; van Noordwijk and van Schaik 1987). At the same time, they also show marked intraspecific (between groups, but also across individuals within the same group) variation in social behaviour, specifically in the patterning and distribution of agonistic and affiliative social interactions among individuals (Anuar and Ullah 2011; Marty et al. 2019; Sterck and Steenbeek 1997; van Noordwijk and van Schaik 1987). This in turn culminates in marked differences between individual macaques in their social status or dominance ranks, and their connectedness to other macaques within their affiliation networks (Balasubramaniam et al. 2020; Marty et al. 2019, 2020; Sterck and Steenbeek 1997; van Noordwijk and van Schaik 1987). Across their range, long‐tailed macaques also show dietary behavioural flexibility preferring both natural and human‐provisioned or anthropogenic food, the access to which might also be strongly influenced by their social status (Marty et al. 2020). For these reasons, long‐tailed macaques are considered an ‘edge’ or (peri)urban wildlife species that overlap spatially and interact socially with humans and anthropogenic landscapes across many parts of their natural range (Gumert 2011).

To‐date, much of our knowledge of long‐tailed macaque microbiomes has emerged from research on captive populations, and in the context of understanding links between tuberculosis (Tb: *Mycobacterium* spp.) infectious stages and corresponding shifts in macaque microbiomes that are indicative of dysbiosis (Sawaswong et al. 2021, 2023). In comparison, our knowledge on the community ecology of the microbiomes of free‐living or wild long‐tailed macaques is very limited. In a previous study, we revealed that social grooming among long‐tailed macaques was linked with shared gut microbiota, but also dominance rank influenced macaques' overall microbial diversity through mediating their access to anthropogenic food (Kesh et al. 2026). As such, our study complements and adds to these findings by taking a community ecology approach.

Figure 1 shows the graphical abstract of our study. Among a group of wild long‐tailed macaques, we (1) assessed whether gut microbiota exhibit non‐random patterns in community structure—that is, whether they display nested structures that are indicative of widespread abundance of several ‘core’ bacterial taxa across many macaques, versus modular or at the extreme segregated structures characterised by the sub‐grouping of specific bacterial taxa among specific macaques. In metacommunity ecology, ‘nestedness’ refers to a pattern that is reflective of a ‘diversity gradient’, that is, where entities (individual animals, populations, locations or sites) with lower species richness are subsets of entities with higher species richness. Thus, we asked whether the distribution of bacterial taxa across individual macaque hosts followed a similar gradient of species richness. Specifically, we sought to determine if macaques with lower microbial diversity hosted bacterial communities that were a subset of those found in macaques with higher diversity (Balasubramaniam, Muller‐Klein, et al. 2025; Balasubramaniam, Navarrete Macias, et al. 2025; Fountain‐Jones et al. 2019; Strona and Veech 2015). Alternatively, we considered whether the microbial communities exhibited modular patterns, or in an extreme case, segregated patterns wherein specific bacterial taxa would be associated with a subset of macaques, or even exclusively within individual macaques, suggesting the presence of separate subgroups of microbial communities rather than a continuous gradient (Fountain‐Jones et al. 2019; Strona and Veech 2015). By determining whether bacterial taxa showed such nonrandom patterns of relative abundance across macaques, we aimed to establish a premise for expecting bacterial community structure to be shaped by deterministic factors rather than solely by stochastic processes (Zhou and Ning 2017; Hayashi et al. 2024).

**FIGURE 1:**
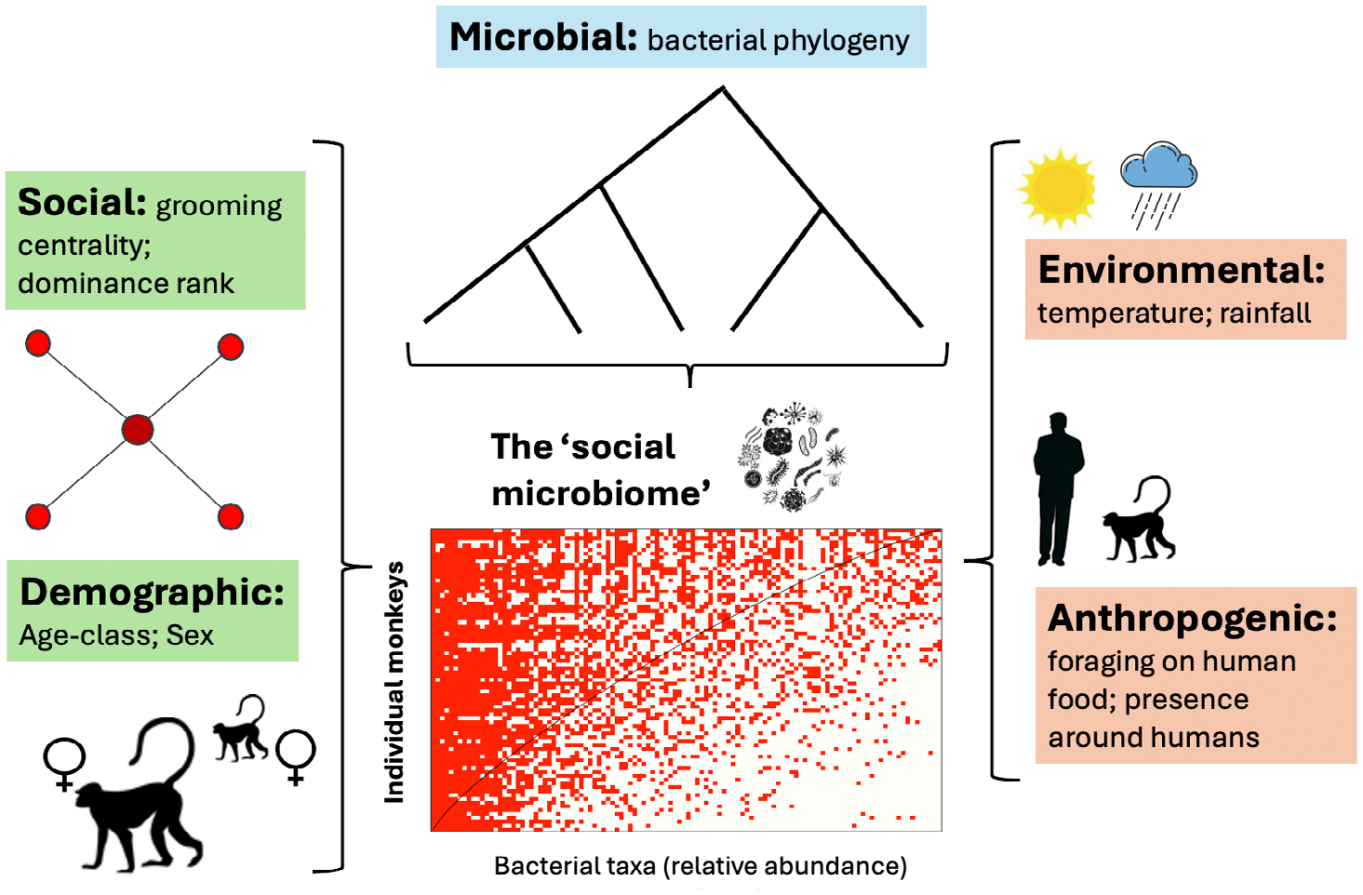
Aims of the study.

Following this, we (2) assessed the impact of inter‐individual differences in macaque sociality on the overall variance in the relative abundance of gut microbiota. As indicators of macaques' sociality, we evaluated individuals' dominance rank as a measure of their ‘social status’, and their connectedness or centrality within their social grooming networks as a measure of their ‘social integration’ within their group. Thus, we evaluated the relative impact of these two aspects of macaque sociality, compared to the effects of macaques' demographic characteristics (age‐ and sex‐class), exposure to anthropogenic factors (foraging on anthropogenic food, using or in direct contact with anthropogenic surfaces: see Section 2 for definitions), and exposure to abiotic environmental factors (temperature, rainfall), on bacterial abundance. Next, we (3) determined which taxa were positively (versus negatively) impacted by rank or centrality, and thereby showed a greater likelihood of occurring or being more abundant in more socially stronger or integrated macaques. Finally, we determined whether (4) specific taxonomic units of bacteria seemed to form strong associations within hosts, that is, systematically co‐occurred within the same macaque hosts even after accounting for the effects of social, abiotic or anthropogenic environmental factors on bacterial abundance.

## 2 Methods

### 2.1 Study Site and Subjects

We studied a single group of long‐tailed macaques at Wat Khao Thamon, located in the Phetchaburi province of Thailand (13°02′ N, 99°57′ E). The region is an anthropogenic landscape which includes a Buddhist temple, surrounded by agricultural rice fields, artificial ponds and villages, with a few ‘natural’ patches of sub‐tropical forests. There are multiple (4+) groups of long‐tailed macaques with overlapping home ranges within the site, with around 400 individuals reported in 2021 (Aiempichitkijkarn et al. 2025).

For this research, we studied the largest group at the study site, composed of approximately 123 adults and 50 juveniles. This group has been followed for extended behavioural research since 2021 (Aiempichitkijkarn et al. 2025), during which time they have been spending a large proportion of their time near the Buddhist temple and the surrounding regions of the study site described above. They were therefore well‐habituated to humans and were regularly provisioned with anthropogenic food by the temple monks and occasionally received provisioned food from temple visitors. They could also be easily followed across most parts of their range by researchers to record continuous behavioural observations. The majority of individuals had already been identified during a previous study (Aiempichitkijkarn et al. 2025), as well as for a recently commenced long‐term study of long‐tailed macaque social microbiomes (Kesh et al. 2026; this article).

### 2.2 Behavioural Data Collection

Behavioural data were collected by two field observers TH and SP for a 6‐month period, from April to September 2025. Prior to this, the field observers completed inter‐observer reliability tests with each other and independently with KB within a one‐month preliminary training phase (Cohen's Kappa with a cut‐off of > 0.85: Martin and Bateson 2007; Kaburu et al. 2019). Subjects were adult male and female macaques; considering time and budgetary constraints we ended up collecting data on 75/123 (or 61% of all) adults ensuring that these represented an even distribution of macaques' demographic (age and sex) classes and dominance ranks (as predetermined in our previous study: Aiempichitkijkarn et al. 2025). Data were collected for 4 days a week, across two sessions with the first being in the morning (7 AM to 11 AM) and the second in the afternoon (1 PM to 5 PM). All behavioural data were entered directly into Samsung Galaxy tablets, using customised forms and databases generated using the HanDBase software (details in Kaburu et al. 2019; Aiempichitkijkarn et al. 2025).

For this study, two major types of behavioural data were recorded. First, a continuous 10‐min *focal animal sampling* with *point‐time scan sampling* embedded within each focal session (Altmann 1974; Martin and Bateson 2007). On each day, a pre‐determined, randomised order was used to find focal individuals, ensuring that the same animal was never observed more than once on the same day. Within a focal session, all macaque‐macaque and human‐macaque social interactions were recorded in a continuous manner. Specifically, these included the following:

- Agonistic interactions, specifically either aggression (threat, lunge, chase, slap and attack) that was followed by a submissive signal (displacement, avoidance, fleeing, silent bare‐teeth, scream), or just a submissive signal to a non‐aggressive or neutral (e.g., approach) behavioural context;
- Macaque‐macaque social grooming, that is, cleaning or manipulating the fur of another individual (Henzi and Barrett 1999; McFarland 2018) where the focal animal was seen grooming, or being groomed by, another individual who was also one of the focal animals. For these we additionally recorded the start and end times of each grooming bout, to later calculate the proportions of time spent by animals engaging in grooming;
- Point‐time scan of main activity, that is, pausing continuous focal sampling once every 2 min to record the focal animal's main activity (Resting, Locomotion, Self grooming, Grooming, Socialising, and whether it was foraging on natural food or on anthropogenic food) along with the location or surface (‘natural’ if the macaques were found in spaces that were forested rather than human‐made, ‘anthropogenic’ if they were found on human‐made surfaces like buildings, roads, paved areas, fences, etc.) on which it was present when scanned.

Second, focal animal sampling temporarily ceased in favour of conducting *all occurrences* event sampling whenever the group was provisioned by monks, other temple staff or visitors (Kaburu et al. 2019; Martin and Bateson 2007). This session lasted for 30 min each. They were conducted only if a provisioning event was observed and were recorded no more than twice on the same day. The location of provisioning, food type and provisioner identity were recorded. Following this, instances of macaque‐macaque agonistic interactions that involved at least one of the 75 focal animals (aggression followed by submission, or just submission in a non‐aggressive or neutral context) were recorded in a sequential manner, by systematically scanning the location from left to right. For full details and definitions of the above‐described behaviours, see previous research (Aiempichitkijkarn et al. 2025; Balasubramaniam et al. 2020; Kaburu et al. 2019; Marty et al. 2019, 2020).

### 2.3 Macaque Social Behaviour, Demographic Characteristics and Exposure to Anthropogenic Factors

We estimated macaques' dominance ranks from a dyadic dominance wins‐and‐losses matrix in which the cells contained frequencies of submissive signals (both following aggression and in a neutral or non‐aggressive context) shown by animals in the columns (losers) towards animals in the rows (winners) (details in Kesh et al. 2026). We included all focal individuals, both males and females, into a single such wins‐and‐losses matrix. On this matrix, we implemented the Elo‐rating approach (Neumann et al. 2011) using the *EloRating* package (Neumann and Kulik 2014) in R to estimate dominance ranks. This method is commonly used by animal behaviourists and indeed was found to generate rank orders that were also strongly correlated with rank orders generated by other popularly used cardinal ranking methods of David's scores based on the *D*_*ij*_ and the *P*_*ij*_ indices (Gammell et al. 2003; de Vries et al. 2006; Leiva and de Vries 2010) (Spearman's rho > 0.9, *p* < 0.001; see Kesh et al. 2026 for more details on these calculations).

From the focal animal sampling, we extracted a three‐column edge‐list to construct and conduct Social Network Analysis (SNA), using the package *iGraph* (Antonov et al. 2023; Csárdi et al. 2026) in R (R Core Team 2025). This constituted pairs of macaques, that is, the focal individual and another focal individual, that had engaged in social grooming, with the values of cells in the third column containing information on macaques' proportions of time spent grooming (i.e., the total grooming time between the pair of focal IDs divided by the total focal observation time of the two macaques; more details as well as a social network figure may be found in our other manuscript: Kesh et al. 2026). From this network we calculated the ‘grooming centrality’ or ‘strength centrality’ of each macaque, as the weighted sum of its direct connections within its grooming network (Sosa et al. 2021; Sueur, Jacobs, et al. 2011). The choice of strength over other measures such as betweenness centrality and eigenvector centrality stemmed from the fact that we showed, in our previous study, that macaques' direct connectedness or strength within their grooming networks seemed to have a stronger impact on their overall microbial diversity than either betweenness or eigenvector (Kesh et al. 2026). Furthermore, the choice of social grooming centrality over proximity centrality also stemmed from our previous research which revealed that grooming, presumably on account of involving more and prolonged periods of direct contact, more strongly predicted gut microbial sharing among these macaques compared to social proximity (Kesh et al. 2026).

All focal animals were assigned demographic characteristics based on distinguishing physical features, specifically based on their ‘sex’ (male versus female: using external genitalia and from previous estimations: Aiempichitkijkarn et al. 2025) and ‘age‐class’ based on morphological features like body size, posture at the shoulder, hair colouration and features of the skin (categorised into ‘prime adult’ if broad‐shouldered with fewer wrinkles and darker coloured and shorter hair, and or ‘old or post‐prime adult’ if shoulders were sagging, grey‐to‐white hair colour around the face, and loose or sagging skin).

As measures of macaques' exposure to anthropogenic factors, we estimated each individual's (i) ‘foraging on anthropogenic food’ as the percentage of the total numbers of point‐time scans (S) in which the macaque was observed foraging on anthropogenic food (Fa) (Fa/S). We also estimated macaques' overall (ii) exposure to humans, as the proportion of the total number of scans (S) in which macaques were found on an ‘anthropogenic surface’, that is, had been scanned performing a behaviour (apart from foraging) (so Resting, Locomotion, Self grooming, Grooming or Socialising) while on a human‐made or anthropogenic surface such as on a building, fence, sidewalk, road, etc.

### 2.4 Faecal Sample Collection and Abiotic Environmental Covariates

Faecal samples were collected opportunistically throughout the day by a third observer, from freshly dropped faeces whenever any focal macaque was observed defecating. Caution was taken to collect samples (0.5–2 g) only from the central portion of the dropped faeces, thereby avoiding contact with surrounding soil. Faecal material was picked up using sterile swabs; these were then stored in tubes containing 1–2 mL of NAP (Nucleic Acid Preservation) buffer until they were sent to the laboratory of SP within Chulalongkorn University, Bangkok, for further analysis. The following information was recorded for each sample, on the label and/or directly into a separate database generated within the HandBase app in Samsung Galaxy tablets: the date of sample collection, time of sample collection, GPS location, focal animal ID, animals' age‐class, animal's sex‐class, type of surface (anthropogenic or natural) on to which the sample had been deposited by the defaecating animal, and prevailing abiotic environmental (climatic) conditions of ‘temperature’ (in °C) at the time of collection and ‘rainfall (in mm)’ over the entire day of collection.

### 2.5 Faecal Sample Processing and Microbiome Characterisation

Faecal samples were subject to full‐length 16S sequencing based on Oxford Nanopore Technologies (ONT). Total genomic DNA was extracted using the ZymoBIOMICS DNA Miniprep Kit (Zymo Research, USA) according to the manufacturer's protocol. The full‐length bacterial 16S rDNA (V1–V9 regions) was amplified using primers described in a previous publication (Sawaswong et al. 2024), comprising 5′ nanopore adaptors and 3′ target‐specific sequences: 16S_27F (5′‐TTTCTGTTGGTGCTGATATTGCAGRGTTYGATYMTGGCTCAG‐3′) and 16S_1492R (5′‐ACTTGCCTGTCGCTCTATCTTCCGGYTACCTTGTTACGACTT‐3′). PCR was performed using 10 ng of genomic DNA, 0.25 μM of each primer and KOD One PCR Master Mix (Toyobo, Japan). The thermal profile consisted of 98°C for 30 s, followed by 25 cycles of 98°C for 10 s, 60°C for 10 s and 72°C for 45 s, with a final extension at 72°C for 5 min. Amplicons were subsequently barcoded via a 5‐cycle PCR using the PCR Barcoding Expansion 1–96 (EXP‐PBC096) kit (Oxford Nanopore Technologies, UK) with a 60 s extension time at 72°C. The resulting library was purified using the QIAquick PCR Purification Kit (Qiagen, Germany), quantified with the Quant‐iT dsDNA HS Assay Kit (Invitrogen, USA), and equimolarly pooled. Following size selection with 0.5× Agencourt AMPure XP beads (Beckman Coulter, USA), 1 μg of the barcoded library was ligated with sequencing adaptors (SQK‐LSK114, ONT) and sequenced on an R10.4.1 (Q20+) flow cell using a MinION Mk1C sequencer (Oxford Nanopore Technologies, UK).

Following this, 16S sequences were subject to bioinformatic analysis of gut microbiota. Raw FAST5 files generated by Nanopore sequencing were basecalled using Guppy basecaller v6.1.2 (Oxford Nanopore Technologies, UK) in super‐accuracy (SUP) mode. Initial sequence quality was assessed using MinIONQC (Lanfear et al. 2019), followed by demultiplexing and adapter trimming via Porechop v0.2.4. The resulting filtered reads were processed for clustering, polishing and taxonomic identification through the NanoCLUST (Rodríguez‐Pérez et al. 2021) pipeline, utilising the Blastn algorithm against reference sequences from the RDP database (version 11.5). Bacterial abundance outputs were processed in QIIME2 v2021.2 (Bolyen et al. 2019) to generate a taxon table that constituted 119 bacterial ASVs collapsed to the species level, and for which relative abundance of each taxa within each sample/macaque were calculated and used in data analysis.

### 2.6 Data Analysis

All data analyses were restricted to *n* = 75 individuals in the group, for which we had collected and processed both behavioural and microbiome data. To investigate whether bacterial taxa among macaques exhibit non‐random patterns of nestedness, modularity or segregation among hosts, we used a bipartite network analysis (adapted from Strona and Veech 2015). Using the R package *Bipartite* (Dormann et al. 2014), we constructed ‘*m* × *n*’ networks, connecting one set of nodes containing individual macaques (*m* = 75) to another set of nodes representing bacterial taxa (*n* = 119). We thus constructed two bipartite networks, a ‘weighted network’ in which the edges (values in the cells of the *m* × *n* matrix) were weighted by the relative abundance of taxa within macaques, or an ‘unweighted network’ in which cells essentially only contained information on whether (1) or not (0) a bacterial taxa was present in a particular macaque. For these networks we calculated degrees of nestedness using the R package *vegan* (Oksanen et al. 2013). Using the weighted bipartite network, we applied the ‘nestedNODF’ function to calculate weighted (wNODF) indices of nestedness for bacterial taxa among macaques. On the unweighted matrix, we also used the function ‘nestedtemp’ to calculate the bipartite matrix temperature (Temp) as an indicator of the degree of deviation from entropy or randomness in the distribution of links between macaques and bacterial taxa. For both measures, we also used the ‘oecosimu’ function to perform permutation‐based significance tests comparing observed values to a distribution of wNODF and Temp values generated after 1000 simulations of randomly swapping matrix (network) rows and columns.

To test our remaining aims and predictions, we ran a JSDM using the Hierarchical Modelling of Species Communities (*Hmsc*) package in R (Tikhonov et al. 2020). For this we first input the weighted, bipartite *m* (75 macaques) × *n* (119 bacterial taxa) network of relative bacterial taxa abundance values, as the outcome matrix. As animal‐specific covariates, we input macaques' social variables (dominance rank, grooming strength), demographic characteristics (age‐class, sex), exposure to anthropogenic factors (foraging on anthropogenic food, surface of sample collection), and environmental exposure (temperature, rainfall) (see definitions above). We fitted the model with a Bayesian inference criterion using the ‘normal’ distribution function. We used a default prior and ran two chains of 1500 iterations each, discarding the first 500 as burn‐in and thinning the remainder to obtain 1000 posterior samples per chain per model. We used visual inspection of the MCMC plots and calculations of the Gelman‐Rubin diagnostics to assess model convergence.

To determine the relative effects of macaques' sociality compared to their demographic characteristics, anthropogenic factors and environmental variables (2), we extracted the relative variance contributions of the covariates from the JSDM output, and compared their influence on the observed distribution of relative bacterial taxa abundance. Furthermore, we ran a General Linear Mixed Model (GLMM) with a Beta distribution function using the package *glmmTMB* package in R (Brooks et al. 2017), to determine the impact of covariate type as the independent variable (social vs. demographic vs. anthropogenic vs. environmental), on the outcome of variance proportions contributions. ‘Bacterial taxa’ was included as a random effect, to account for repeated measures of each covariate type on each of the 119 taxa.

To assess which specific taxa were positively versus negatively impacted by macaques' dominance ranks and by their grooming strength centrality (3), we extracted a heat map from the JSDM output, of the predicted probabilities based on the effect sizes (*B* coefficients) of these two aspects of sociality on the relative abundance of each of the 119 taxa. From these, we deciphered how many taxa were significantly positively, versus negatively, impacted by macaques' dominance ranks and grooming strength, that is, tended to be more abundant among higher‐ranking, more central or well‐connected macaques, versus among lower‐ranking, less central or more peripheral macaques.

Finally, to determine whether bacterial taxa formed stable associations or co‐occurred within macaque hosts independent of the effects of host or covariates (4), we extracted a ‘residual’ taxa‐taxa bacterial co‐occurrence matrix of positive and/or negative taxa‐taxa associations from the JSDM output. To determine whether bacterial phylogenetic relationships predicted taxa‐taxa co‐occurrence more strongly than host‐related factors like macaques' age‐class or sex‐class, we ran a univariate Matrix Regression using a Quadratic Assignment Procedure (MR‐QAP: Dekker et al. 2007; Farine and Carter 2022), using the *sna* R package (Butts 2008). In this test the outcome matrix was the bacterial co‐occurrence matrix, in which the cells contained values of the co‐occurrence correlation coefficients between taxa in the rows and taxa in the columns. As a predictor, we included a matrix of taxa‐taxa phylogenetic distances extracted from the bacterial phylogenetic tree. All statistical tests were two‐tailed, with the alpha value set at 0.05.

## 3 Results

Rarefaction curves confirmed that sequencing depth was sufficient to capture the richness of the 119 bacterial taxa across all 75 macaques (Figure S1). The 119 bacterial taxa identified were classified into 69 genera and 9 phyla. The Relative bacterial abundance was strongly dominated by two phyla, Firmicutes (mean 63.9% ± 7.9% SD) and Bacteroidetes (28.0% ± 6.5%), which together accounted for 91.8% of total relative abundance; this was followed by Proteobacteria (2.9% ± 3.6%), Actinobacteria (2.2% ± 4.4%) and five minor phyla (Spirochaetes, Verrucomicrobia, Lentisphaerae, Fibrobacteres and Tenericutes), each contributing < 1.4%. At species‐level resolution, the most abundant taxa, *Oscillibacter valericigenes* (7.8% ± 4.2%) and *Prevotella copri* (7.8% ± 6.3%), were closely followed by *Intestinimonas butyriciproducens* (4.7% ± 2.7%) and *Faecalibacterium prausnitzii* (3.8% ± 2.8%) (Figure S2). Prevalence across the group also varied widely: three taxa ( *Prevotella copri* , *Faecalibacterium prausnitzii* and *Dorea longicatena* ) were detected in all 75 macaques, whereas the median taxa was detected in only 54.7% of individuals (Figure S3).

1. Gut bacterial communities showed nested rather than modular or segregated architectures:

The bipartite network analysis confirmed that gut bacterial taxa among long‐tailed macaques showed non‐random patterns of abundance. Bacterial taxa associated with individual macaques exhibited strongly nested rather than modular or segregated structures (Figure 2). This is supported both by the significantly high scores of weighted NODF (wNODF = 44.85, *p* < 0.01) and nestedness temperature (Temp = 39.21, *p* < 0.01). In other words, while some ‘core’ bacterial taxa were present in many or the majority of macaques, other bacterial taxa were found only within fewer or only a minority of macaques (Figure 2).

**FIGURE 2:**
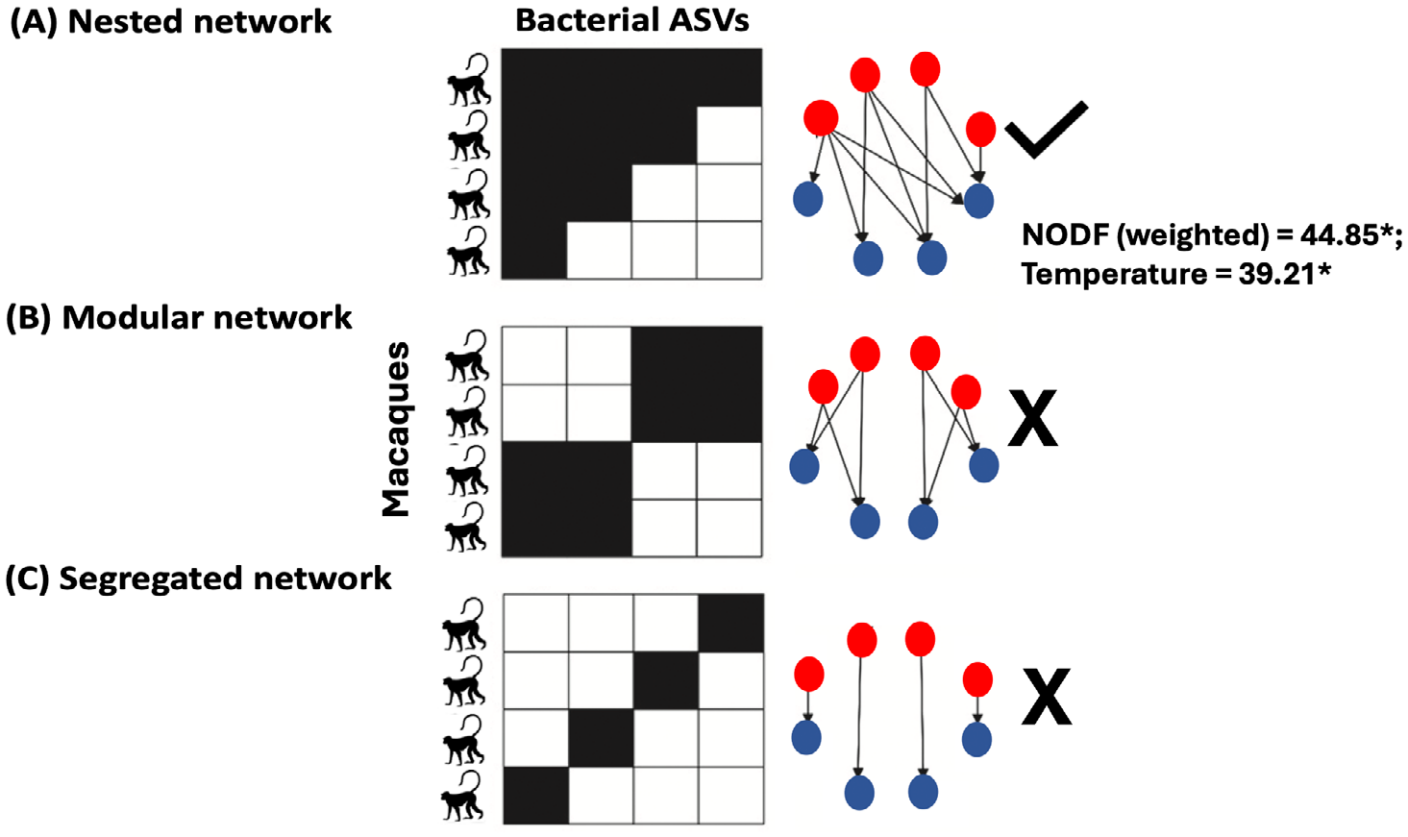
Bipartite networks indicating possible patterns of the relative abundance of bacterial taxa that constitute the gut microbiome across macaques. Bipartite network analysis showed strong support for a (A) nested pattern, that is, of many bacterial taxa being abundant across most or all of the macaques, while just a few (minority) of taxa were found only in a few macaques. On the other hand, there seemed little or no evidence for (B) modular or (C) segregated communities, that is, of specific bacterial taxa only occurring in specific macaques, or even in specific individuals, but not so in other macaques (adapted from Strona and Veech 2015).

2 Individuals' social behaviour showed the strongest effects on microbiome assembly, followed by demographic characteristics, anthropogenic factors and abiotic environmental variables:

The beta distribution GLMM showed that the mean variance contribution of social behavioural factors to explain the observed taxa abundance was significantly greater than the variance contributions of anthropogenic (*z* = 3.36, *p* < 0.01) and abiotic environmental (*z* = 3.25, *p* < 0.01) factors (Figure 3; Table S1). Moreover, social behaviours also showed a nonsignificant trend for having a greater effect than demographic characteristics on taxa variance contributions (*z* = 1.65, *p* < 0.10; Figure 3; Table S1).

**FIGURE 3:**
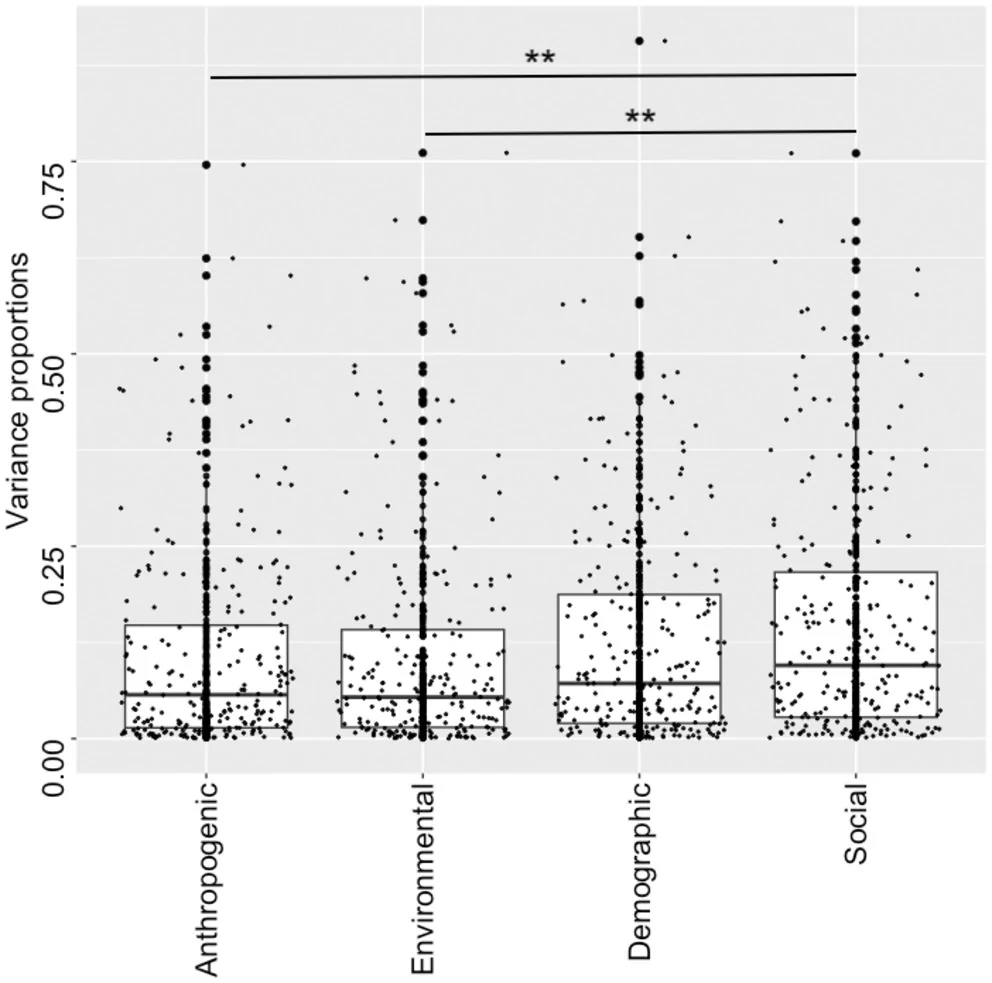
Boxplot of the result of the beta‐distribution GLMM which tested the relative contributions of macaques' social behaviour (dominance rank, grooming strength centrality), demographic characteristics (age‐, sex‐class), exposure to anthropogenic factors (foraging on anthropogenic food, exposure to humans), and abiotic environmental factors (temperature, rainfall), to the observed variance proportions (*Y*‐axis) of relative bacterial taxa abundance as estimated from the JSDM simulation run. Comparisons that were statistically significantly different are indicated (**p < 0.01).

Furthermore, comparisons of aspects of macaque sociality accounted for the largest (31%) proportion of variance in bacterial abundance (caterpillar plot of Figure 4). This was followed by demographic characteristics (26%), and finally by biotic environmental (22%) and anthropogenic (21%) factors. Among the social factors, macaques' dominance rank (16%) and grooming strength centrality (15%) contributed nearly equally to the explanation of bacterial abundance (Figure 4).

**FIGURE 4:**
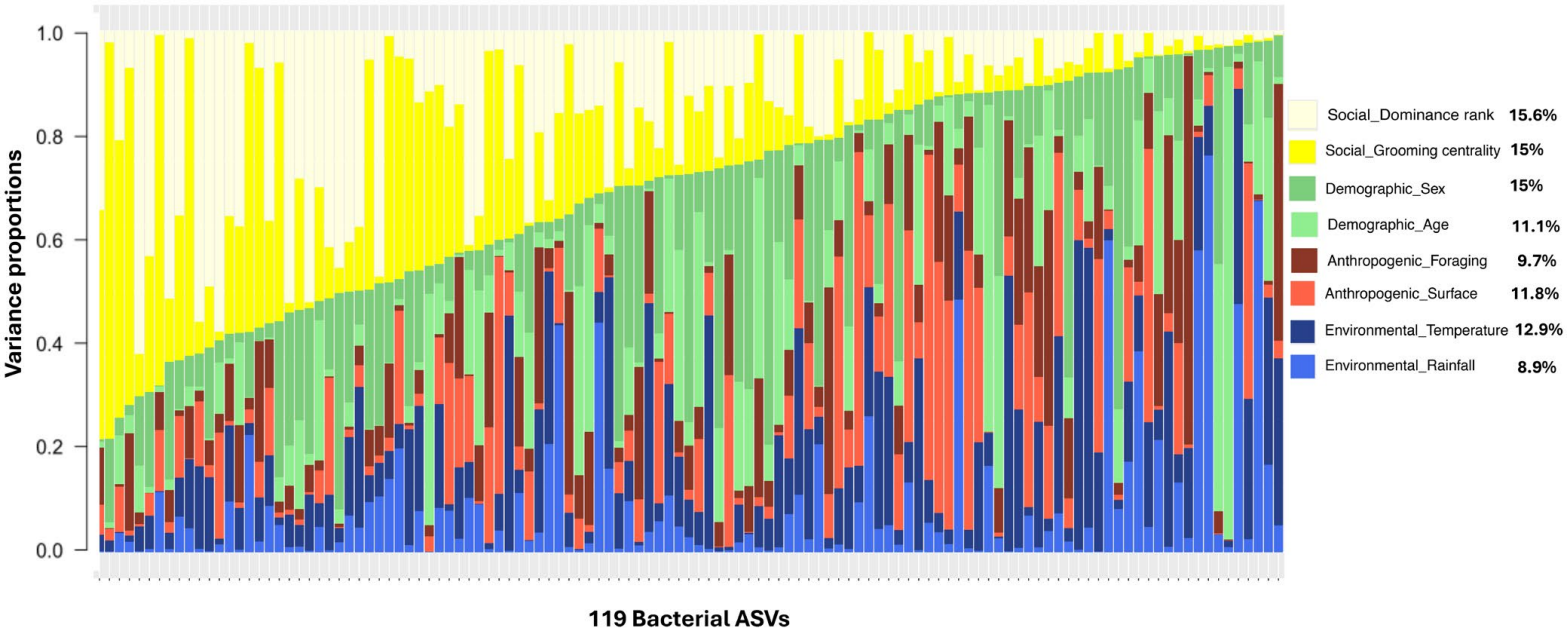
Caterpillar plot of variance contributions explained by the JSDM, indicating the relative contributions of social behavioural (dominance rank, grooming strength centrality), animal‐specific demographic (age‐, sex‐class), anthropogenic (foraging on anthropogenic food, exposure to humans) and abiotic environmental (temperature, rainfall) covariates to the relative abundance of 119 bacterial taxa. Values next to each covariate indicate the mean percentage contribution of that covariate, for example, dominance rank contributed to ~16% of the overall proportion of the observed variance in bacterial abundance. Bacterial taxa (*X*‐axis) are ordered in the ascending order of the total percentage contribution of macaques' social behavioural covariates (dominance rank + grooming centrality).

3 Social behaviours showed positive correlations with the abundance of some taxa and negative correlations with the abundance of others:

We found that aspects of macaque social behaviour, both dominance rank and grooming centrality, significantly impacted the relative abundance of the majority (117/119, or ~98%) of bacterial taxa. Figure 5 shows a ‘heat map’ from the JSDM output, of the impact of macaques' social factors—dominance rank and/or social grooming connectedness—on the relative abundance of each of the 119 taxa. Coloured cells indicate that, across the 75 macaques, dominance rank and grooming strength centrality showed either a significantly positive (cells in red) or a significantly negative (cells in blue) correlation with the relative abundance of 97/119 or ~81.5% of taxa (dominance rank) and ~102/119 or ~86% of taxa (grooming centrality). More specifically, we identified 45 taxa that were significantly positively associated with dominance rank, that is, were found more in high‐ranking compared to low‐ranking animals, and 52 taxa that showed the opposite effect, that is, were more abundant among low‐ranking compared to high‐ranking macaques. Likewise, 53 taxa showed a significant, positive correlation with grooming centrality, that is, were more abundant in socially central compared to socially peripheral macaques. On the other hand, 49 taxa showed significant, negative associations with grooming centrality, that is, were more abundant among socially peripheral compared to central macaques. A high proportion (89/119 or ~75%) of taxa tended to be more abundant in *either* high ranking *or* more highly central individuals.

**FIGURE 5:**
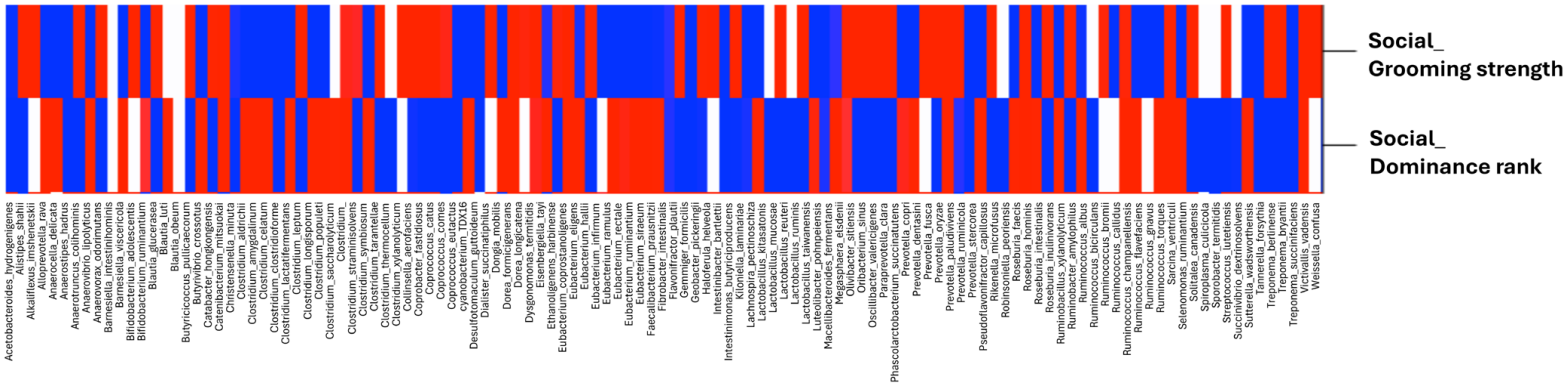
Heat map indicating the effects of macaque social behavioural covariates, particularly dominance ranks and grooming social network centrality, on the relative abundance values of each of the 119 bacterial taxa. Cells in red indicate significantly positive correlations, and in blue significantly negative correlations, between the behavioural covariates (rank or centrality) and the corresponding taxa. Blank cells indicate no significant correlation.

4 ASV‐ASV co‐occurrence among macaques is strongly influenced by bacterial phylogeny:

Figure 6 shows the ‘residual’ bacterial co‐occurrence matrix or network extracted from the JSDM run. As such, the co‐occurrence network revealed that several taxa pairs (2001/7021, or 28.5%) showed significantly positive tendencies to co‐occur within the same macaques, while fewer pairs (1219/7021, or 17.4%) also showed significantly negative tendencies to co‐occur within macaques. Furthermore, the MR‐QAP test showed that this bacterial co‐occurrence network was strongly predicted by bacterial phylogenetic relatedness. Specifically, distantly related taxa, that is, that were farther apart in the phylogenetic tree, were more likely to co‐occur within the same host than more closely related taxa (*B* = 0.06, *p* < 0.01; *F*(df = 7019) = 62.14, *p* < 0.01). Furthermore, these effects of bacterial associations or co‐occurrence on community composition were independent of the impact of social, demographic, anthropogenic and environmental covariates on bacterial relative abundance.

**FIGURE 6:**
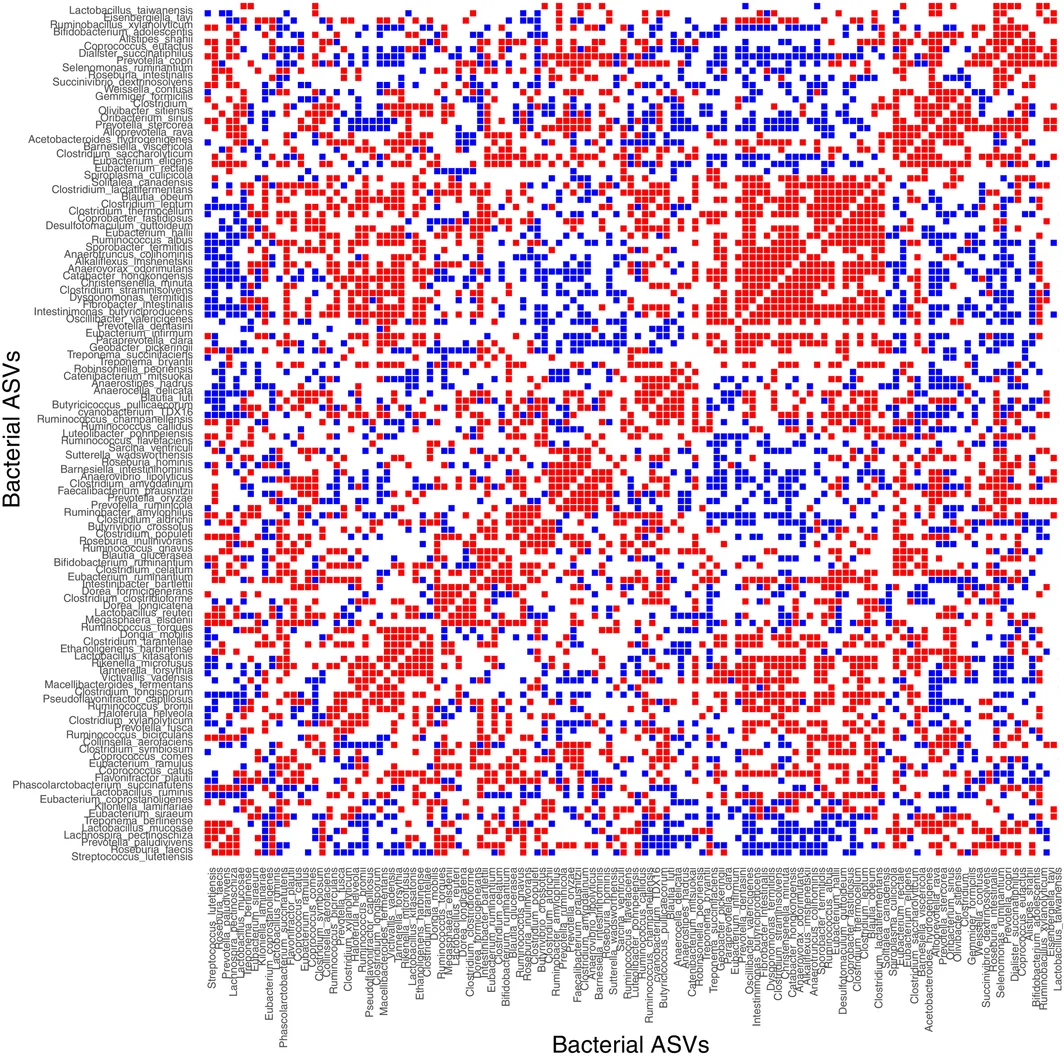
Co‐occurrence matrix of bacterial taxa‐taxa associations extracted as residual values from the JSDM simulation run, after accounting for the effects of macaques' social behaviour, demographic characteristics, abiotic environmental factors and anthropogenic factors, on relative bacterial abundance. Rows and columns indicate that the 119 bacterial taxa isolated for this study. Coloured cells indicate significantly positive (red) or significantly negative (blue) taxa‐taxa co‐occurrence correlation coefficients.

## 4 Discussion

We addressed a critical gap in our understanding of the evolutionary ecology of animals' ‘social microbiomes’, that is, the impact of animal sociality and group living on their gut bacterial composition and community assembly (Debray et al. 2024; Sarkar et al. 2020). Traditional empirical assessments of the social microbiome concept have assessed how bacterial communities are embedded within scales or levels of host social organisation, that is, the assortment of individual animals into social groups and populations, and indeed their composition and sizes (reviewed in Debray et al. 2024; Sarkar et al. 2020). Here we build on these efforts by assessing how inter‐individual differences in social behaviour affect microbiomes, among wild but peri‐urban long‐tailed macaques that live in complex social groups and under increasing degrees of exposure to human (anthropogenic) impact. We found that the emergent outcomes of macaques' agonistic and affiliative social interactions, specifically their dominance ranks and social grooming network connectedness or centrality, had a stronger impact on bacterial relative abundance than macaques' demographic characteristics or their exposure to anthropogenic factors in the environment. Below we discuss our findings in depth and their wider implications in terms of our current and prospective understanding of wildlife microbiomes in a changing, human‐impacted world.

The bipartite network analysis showed that bacterial communities in long‐tailed macaques exhibited nested rather than segregated or modular structures. That is, many bacterial taxa were present in nearly all the macaques sampled in the group, while only a few taxa appeared in fewer macaques. This suggests that while many macaques showed high microbial diversity, at least some macaques had lower microbial diversity consisting of only a fraction of bacterial genera that constituted the overall bacterial community diversity. From a bacterial community ecology perspective, this finding provides crucial empirical support for the idea of ‘core’ versus ‘non‐core’ bacteria, that is, those taxa that are found across the majority of hosts in a group or population versus (in this case) a minority of taxa whose occurrence may more dynamically respond to transient environmental factors (Balasubramaniam, Muller‐Klein, et al. 2025; Grieneisen et al. 2017; Risely 2020). Furthermore, the clear architecture of long‐tailed macaque microbiomes further strengthened our premise, that bacterial assembly and relative abundance among long‐tailed macaques are strongly shaped by deterministic factors rather than merely being chance outcomes of stochastic processes (Kesh et al. 2026; Hayashi et al. 2024; Zhou and Ning 2017).

The JSDMs showed that aspects of macaque social behaviour, specifically their dominance ranks and their connectedness within their contact social grooming networks, had the strongest effects on the observed variance in relative bacterial abundance. This finding lends strong empirical support to the ‘social microbiome’ concept, that is, to the idea that aspects of animal sociality and (here) their social behaviour can have a strong impact on gut microbiomes despite the effects of other animal‐specific, environmental and microbe‐specific factors. It thus builds on previous findings in important ways. Primarily, JSDMs advance previous empirical research on social microbiomes, that have predominantly focused on establishing links between animals' social network connectedness and their overall degrees of bacterial (dis)similarity (e.g., Heidrich et al. 2025; Raulo et al. 2021; Tung et al. 2015; reviewed in Debray et al. 2024). Indeed, many of these studies have explicitly emphasised and implemented SNA tools to test evidence for the social transmission of microbes. On the other hand, JSDMs account for the fact that microbial acquisition may be influenced by multiple animal‐specific and environmental factors, or indeed by microbe‐specific characteristics such as taxonomic category, phylogenetic relatedness or even microbe‐microbe within‐host ecological associations independent of phylogeny (reviewed in Balasubramaniam, Muller‐Klein, et al. 2025; Balasubramaniam, Navarrete Macias, et al. 2025). Furthermore, rather than composite indicators of overall microbial (alpha or beta) diversity, similarity, or relative abundance, the JSDM approach evaluates the relative effects of animal‐specific and environmental covariates on the relative abundance of individual bacterial taxonomic units (here, taxa) that constitute the microbiome (Balasubramaniam, Muller‐Klein, et al. 2025; Balasubramaniam, Navarrete Macias, et al. 2025). Such evaluations of bacterial communities as complex, multivariate assemblages rather than as composite or univariate entities accommodate the real possibility that sociality, rather than merely contact‐mediated sharing, can have highly variant and even contrasting effects on the acquisition and abundance of different bacterial taxa.

We found that dominance rank and grooming network centrality impacted different bacterial taxa in different, sometimes contrasting ways. While some bacterial taxa that have been linked to reduced inflammation and improved intestinal gut health (Parker et al. 2020; Yang et al. 2025) were more abundant among high‐ranking (e.g., Alloprevotella, Blautia, Eubacterium) or more central individuals (e.g., Prevotella, Roseburia, Weissella), other beneficial bacteria were also were more abundant among low‐ranking (e.g., Allistipes, Coprococcus, Flavonifractor) or less well‐connected (e.g., Eubacterium, Prevotella, Sutterella) macaques. Previously, comparative studies of macaque sociality have reported marked, inter‐ and intraspecific variation in the extent to which dominance ranks are correlated to individuals' social network centrality. In general, this correlation is expected to be higher among species such as rhesus macaques and long‐tailed macaques that show greater degrees of despotism, wherein high‐ranking individuals also tend to be the most attractive grooming partners (Balasubramaniam et al. 2018; Sueur, Petit, et al. 2011). However, this was not the case in our group—we found only a moderate‐to‐low correlation between rank and centrality (Pearson's *R* = 0.18, *p* = 0.12). This suggests that social behavioural benefits such as grooming may be more evenly distributed across the whole group. This is also reflected in macaques' microbiome acquisition, in terms of individuals standing to gain several complementary bacterial taxa with known health benefits, irrespective of their rank or their social connectedness, or specifically even if they were low‐ranking or socially peripheral within their group. Future research should therefore look to disentangle the *processes* that underlie such differential microbial taxa acquisition, for instance bacterial acquisition on account of inter‐individual variation in physiology, genetic relatedness and/or diet that might be linked to variation by rank or centrality, or indeed the potential sub‐grouping of macaques by matriline or (closer compared to more distant) rank‐differences. Moreover, whether such acquisition of different, advantageous taxa on account of individuals' rank or centrality culminates in discernible health‐ or fitness benefits to macaques irrespective of their rank or their grooming centrality remains to be investigated.

In comparison to macaque sociality, demographic characteristics, specifically macaques' age‐class and sex, had a slightly weaker impact on bacterial assemblages. Over the past two decades, long‐term behavioural studies on a wide range of social taxa have established how the strength and diversity of animals' social connections change with age (Siracusa et al. 2022), or indeed how factors like kin‐related inheritance and/or sex‐specific dispersal or immigration might influence dynamic changes to dominance ranks (reviewed in Clutton‐Brock and Sheldon 2010; Clutton‐Brock 2021). Therefore, it is conceivable that macaques' age‐class and sex may impact microbiomes more so via influencing their social behaviour. Indeed, our previous study on this group revealed how macaques' age‐class strongly interacted with dominance ranks to influence inter‐individual differences in overall microbial diversity and the relative taxonomic abundance of Firmicutes and Bacteroidetes phyla that are critical to maintaining gut health and homeostasis (Kesh et al. 2026). As such, testing for such interactive effects of social and demographic characteristics on bacterial assemblages was beyond the scope of this study and is a noteworthy limitation of JSDM approaches more generally (Balasubramaniam, Muller‐Klein, et al. 2025; Balasubramaniam, Navarrete Macias, et al. 2025; Fountain‐Jones et al. 2019).

Relative to macaque sociality and demographics, exposure to abiotic and anthropogenic environmental factors showed significantly weaker effects on relative bacterial abundance. One reason for this may have to do with the spatiotemporal scale of sample collection; rather than shorter time‐frames (as in this study), it is likely that the effects of environmental factors such as temperature and rainfall on microbiomes may be more discernible when behavioural and microbial data are collected across longer time‐scales spanning multiple years or seasons. Regarding macaques' exposure to anthropogenic factors, all macaques within our study group had access to, and predominantly foraged on, human‐provisioned food at the Buddhist temple within their home range (Aiempichitkijkarn et al. 2025; Kesh et al. 2026). Rather than inter‐individual differences in the time spent consuming the same types of food, wildlife microbiomes may more likely be influenced by the time elapsed between when an animal was last seen foraging and when its sample was collected (e.g., Risely et al. 2022, 2023; Balasubramaniam, Muller‐Klein, et al. 2025), by longer‐term, seasonal or spatiotemporal variation in (natural or anthropogenic) resource availability and consumption (e.g., Ingala et al. 2019; Sun et al. 2016), or by spatiotemporal variation contact‐ and non‐contact spatial or social interactions with humans, feral animals or livestock (Fackelmann et al. 2021). Thus, we speculate that anthropogenic impacts on macaque microbiomes, rather than between individual animals exposed to broadly similar degrees of human impact, may be clearer across other spatial or social organisational scales, for instance across multiple groups of macaques in the Phetchaburi region or across Thailand living across an anthropogenic gradient defined by varying degrees of overlap with humans and domestic animals, and thereby also by inter‐individual or inter‐group diversity in macaques' foraging ecology and relative degrees of reliance on anthropogenic versus natural food resources (Aiempichitkijkarn et al. 2025; Gumert 2011; Kesh et al. 2026; Malaivijitnond and Hamada 2008).

Finally, we found strong evidence for microbe‐microbe associations within hosts independent of the effects of social, demographic and environmental variables on bacterial assemblages. This was characterised by the ‘residual’ bacterial co‐occurrence network, which showed several taxa pairs that were significantly positively and negatively associated with each other. Moreover, the finding that bacterial co‐occurrence was negatively associated with phylogenetic distances suggested that distantly related bacteria were more likely to co‐occur within the same macaque hosts than more closely related taxa. This finding is consistent with the ecological principles of niche specialisation, habitat filtering or cross‐feeding which would be key for distantly related bacteria to occupy different metabolic niches within the same hosts (Goberna et al. 2019). More definitive conclusions, however, would await molecular ecological studies of the nature and functional significance of taxa‐taxa relationships within hosts. With regard to host sociality, determining the relative importance of phylogenetic relatedness versus aspects of host (macaque) social organisation and social network connectedness or sub‐grouping on bacterial co‐occurrence would provide a much‐needed assessment of the evolutionary ecology of social microbiomes (Sarkar et al. 2024). This would in turn support growing perceptions of microbes as critical, naturally embedded ecosystem components that predominantly form stable interspecies associations with their hosts while only rarely causing dysbiosis or disease risk (French and Holmes 2020; Ladau and Eloe‐Fadrosh 2019).

Our study had some important limitations and considerations. Rather than shotgun metagenomic sequencing which provides higher, strain‐level taxonomic resolution, our microbiome characterisation relied on 16S rRNA to characterise bacterial taxonomic units at the level of the genus. Such lower‐resolution, genus‐level bacterial characterisation limits the ability to definitively identify strain‐level sharing or transmission events. However, identifying transmission events was not a core objective of our study. Indeed, the genus‐level characterisation of bacterial taxa based on 16S sequencing proved sufficient for our objective, that is, for adopting community ecological (rather than epidemiological) approaches to assess the relative importance of deterministic social, environmental and animal‐specific factors, in influencing bacterial relative abundance and co‐occurrence. Moreover, JSDMs are static approaches (Balasubramaniam, Muller‐Klein, et al. 2025; Balasubramaniam, Navarrete Macias, et al. 2025; Fountain‐Jones et al. 2019); they can neither be used to model the sequential order of bacterial transmission events, nor to assess the combined or interactive effects of (for example) social and anthropogenic factors on bacterial acquisition and relative abundance. Previous research on other wild macaque species has revealed how individuals that are more socially peripheral (moor macaques, *Macaca maura* : Morrow et al. 2019), but in other cases also more socially central (bonnet macaques, *Macaca radiata* : Balasubramaniam et al. 2020), tend to be more exposed to humans and anthropogenic factors. So, it is conceivable that macaques' social network connectedness might influence bacterial acquisition via influencing their exposure to anthropogenic factors. Thus, generating and comparing microbiome profiles of humans and other overlapping macaques in the Phetchaburi area, and especially investigating whether macaques' social network centrality influences their acquisition of human‐borne bacteria, would constitute logical next steps.

## Author Contributions

**Krishna Balasubramaniam:** conceptualization (equal), data curation (equal), formal analysis (equal), funding acquisition (equal), investigation (equal), methodology (equal), project administration (equal), resources (equal), software (equal), supervision (equal), validation (equal), visualization (equal), writing – original draft (equal), writing – review and editing (equal). **Jishnu Kesh:** data curation (equal), formal analysis (equal), investigation (equal), methodology (equal), validation (equal), visualization (equal), writing – review and editing (equal). **Nalina Aiempichitkijkarn:** data curation (equal), funding acquisition (equal), investigation (equal), methodology (equal), software (equal), visualization (equal), writing – review and editing (equal). **Robert Atwill:** funding acquisition (equal), investigation (equal), resources (equal). **Suchinda Malaivijitnond:** conceptualization (equal), investigation (equal), methodology (equal), project administration (equal), resources (equal), validation (equal), writing – review and editing (equal). **Brenda McCowan:** conceptualization (equal), funding acquisition (equal), project administration (equal), validation (equal). **Juthamas Nimnual:** data curation (equal), formal analysis (equal), investigation (equal), methodology (equal), validation (equal). **Vorthon Sawaswong:** conceptualization (equal), data curation (equal), formal analysis (equal), investigation (equal), methodology (equal), resources (equal), software (equal), supervision (equal), visualization (equal), writing – original draft (equal), writing – review and editing (equal). **Suthida Visedthorn:** data curation (equal), formal analysis (equal), investigation (equal), methodology (equal), resources (equal), validation (equal), visualization (equal). **Sunchai Payungporn:** conceptualization (equal), data curation (equal), funding acquisition (equal), methodology (equal), project administration (equal), resources (equal), software (equal), supervision (equal), validation (equal), visualization (equal), writing – review and editing (equal).

## Funding

The research was supported by a research ‘Pump Prime’ grant (project code: JC2336) awarded to Dr. Balasubramaniam by the School of Life Sciences at Anglia Ruskin University, Cambridge, UK. It was also supported by discretionary funds awarded to Drs. McCowan and Atwill, within UC Davis.

## Ethics Statement

The protocols used for this research were approved by the Institutional Animal Care and Use Committee (IACUC) of the NPRCT‐CU (approval no. 2075077). All researchers and aspects of research ensured compliance with the legal requirements of Thailand (in particular Phetchaburi province where the research was conducted), which was coordinated through ethics and IACUC approvals and regulations set forth by the NRCT and the DNP in Thailand (Document ID: 893181601753930089; no. 0401/10720). The study underwent ethical and risk‐assessment approvals through research committees within the School of Life Sciences at Anglia Ruskin University (project reference: CAA0/JC2336). Following previous research on the same population, this study also adhered to the American Society of Primatologists (ASP) Principles for the Ethical Treatment of Nonhuman Primates. We followed the ASP Code of Best Practices for Field Primatology. Both field data collectors, employed through NPRCT‐CU, produced updated health certificates as required by their institutional guidelines. They also adhered to strict health and safety protocols, including wearing face masks when in close proximity to their free‐ranging macaque monkey subjects.

## Conflicts of Interest

The authors declare no conflicts of interest.

## Supporting information


**Table S1:** Parameter estimates from the beta distribution GLMM that assessed the impact of major categories of covariates—social behaviour, animal‐specific demographic characteristics, exposure to anthropogenic factors and abiotic environmental variables—on their variance contributions to the observed relative abundance of bacterial ASVs as inferred from the JSDM simulation run.

**Figure S1:** Rarefaction curves for the bacterial taxa across the 75 macaques.
**Figure S2:** Relative bacterial abundance across the 75 macaques summarised at three taxonomic levels. (A) Phylum‐level relative abundance. (B) Genus‐level relative abundance, showing the 15 most abundant of 69 genera individually. (C) Species level relative abundance, showing the 15 most abundant of 119 species.
**Figure S3:** Heat map of relative abundance and prevalence for all 119 bacterial taxa across the 75 macaques (unsorted), ranked from most to least abundant (top to bottom). Per‐sample relative abundance (colour scale, square‐root transformed to improve visibility of low‐abundance ASVs; white = 0%). (B) Prevalence of each ASV, defined as the percentage of the 75 macaques in which it was detected (count > 0).

## Data Availability

The raw sequencing data are available at NCBI Sequence Read Archive (SRA) under bioproject: PRJNA1456312. All data used for this project and R code to replicate our analyses have been made available publicly via figshare (https://doi.org/10.6084/m9.figshare.32333688).
