# Supplementary material for: Social Behaviour Drives Gut Microbiome Community Assembly Among Wild Long‐Tailed Macaques ( Macaca fascicularis ) in Thailand
Source: Ecol Evol. 2026 Nov 1;16(11):e74612. doi: 10.1002/ece3.74612 (PMC13724308; doi:10.1002/ece3.74612)
Supplement: Supplementary file 1 [file ECE3-16-e74612-s001.docx]

**Supporting Table 1: Parameter estimates from the beta distribution GLMM that assessed the impact of major categories of covariates - social behaviour, animal-specific demographic characteristics, exposure to anthropogenic factors, and abiotic environmental variables – on their variance contributions to the observed relative abundance of bacterial ASVs as inferred from the JSDM simulation run.**

|  | **z** | **p** |
| --- | --- | --- |
| (Intercept) | 11.30 | <0.01** |
| Social vs Demographic | 1.65 | 0.1(*) |
| Social vs Anthropogenic | 3.36 | <0.01** |
| Social vs Environmental | 3.25 | <0.01** |
| Demographic vs Anthropogenic | 1.71 | 0.09(*) |
| Demographic vs Environmental | 1.60 | 0.11 |
| Anthropogenic vs Environmental | -0.12 | 0.91 |

**p < 0.01; (*) 0.05 < p < 0.1
