# Supplementary material for: Social Behaviour Drives Gut Microbiome Community Assembly Among Wild Long‐Tailed Macaques ( Macaca fascicularis ) in Thailand
Source: Ecol Evol. 2026 Nov 1;16(11):e74612. doi: 10.1002/ece3.74612 (PMC13724308; doi:10.1002/ece3.74612)
Supplement: Supplementary file 2 [file ECE3-16-e74612-s002.docx]

**Supporting Figure 1:** Rarefaction curves for the bacterial taxa across the 75 macaques.

 **Supporting Figure 2:** Relative bacterial abundance across the 75 macaques summarised at three taxonomic levels. (A) Phylum-level relative abundance. (B) Genus-level relative abundance, showing the 15 most abundant of 69 genera individually. (C) Species level relative abundance, showing the 15 most abundant of 119 species.

**Supporting Figure 3:** Heatmap of relative abundance and prevalence for all 119 bacterial taxa across the 75 macaques (unsorted), ranked from most to least abundant (top to bottom). Per-sample relative abundance (colour scale, square-root transformed to improve visibility of low-abundance ASVs; white = 0%). (B) Prevalence of each ASV, defined as the percentage of the 75 macaques in which it was detected (count > 0).
